# The association of falls with loneliness and social exclusion: evidence from the DEAS German Ageing Survey

**DOI:** 10.1186/s12877-017-0602-5

**Published:** 2017-09-05

**Authors:** André Hajek, Hans-Helmut König

**Affiliations:** Department of Health Economics and Health Services Research, Hamburg Center for Health Economics, University Medical Center Hamburg-Eppendorf, Martinistr. 52, 20246 Hamburg, Germany

**Keywords:** Falls, Loneliness, Social contacts

## Abstract

**Background:**

It remains an open question whether falls are related with social relations, covering subjective (e.g., perceived loneliness) and more objective dimensions (e.g., number of important individuals in regular contact). Consequently, we aimed at examining the association between falls and social ties comprehensively, including loneliness, social exclusion and the number of important people in regular contact.

**Methods:**

Cross-sectional data were used from a population-based sample of community-dwelling individuals aged 40 and over (*n* = 7808) in Germany. Self-rated loneliness was quantified using a short version of the De Jong Gierveld Loneliness Scale. Perceived social exclusion was measured using a scale developed by Bude and Lantermann. Furthermore and in contrast to the subjective outcome measures, the more objective number of important people in regular contact was also used as outcome variable. The experience of a fall in the preceding 12 months (yes; no) was assessed.

**Results:**

Controlling for various possible confounding variables, linear regressions showed that experiencing a fall in the past 12 months was associated with higher social exclusion (β = .08, *p* < .001), and increased loneliness (β = .08, *p* < .001), whereas it was not associated with the number of important people in regular contact.

**Conclusions:**

Findings stress the relation between falls and feelings of loneliness and social exclusion, whereas falls were unrelated to the more objective measure of number of important people in regular contact, suggesting that falls are particularly related to subjective measures of social ties and relations. This underlines the importance of interventions to prevent falls. Preventing falls in turn might help to prevent loneliness and social exclusion.

## Background

Approximately one out of three individuals in old age experienced a fall annually [1], with this proportion increasing with advanced age [2]. The determinants of falls have been widely studied [3, 4] because (1) it is expected that the proportion of older adults will increase and falls are prevalent in old age and (2) falls predict adverse health outcomes, for example, institutionalization, chronic diseases and mortality.

Though the physical injuries caused by the majority of falls are minor, falls often impair functional and mental well-being. They can result in, for example, physical inactivity, reduced self-esteem, decreased health-related quality of life or increased anxiety as well as more depressive symptoms [5–9]. Nevertheless, thus far, few studies have been conducted investigating whether falls are related to aspects of social relations, covering subjective (e.g., perceived loneliness) and more objective dimensions (e.g., number of important individuals in regular contact). For example, a recent cross-sectional study (*n* = 1000, New York City Housing Authority Senior Survey, individuals aged 65 and older via telephone interviews) found that self-reported falls during the past year were neither associated with social contacts (“During the past week, did you talk with relatives, friends, or neighbors on the telephone”) nor with perceived availability of social support (“Is there a friend, relative, or neighbor who could assist you for a few days if necessary?”) [10]. Moreover, a longitudinal study (3 years, *n* = 6692 community-dwelling women aged 70 and over, Study of Osteoporotic Fractures) found that a decreased risk of falls was associated with strong family networks and weak friendship networks [11]. Based on a convenience sample of *n* = 666 community-dwelling individuals aged 50 and over, another study found an association between living alone and experiencing a fall [12]. However, another study [13] found that living alone was inversely associated with falls (*n* = 1573; community-dwelling individuals aged 60 in the Hong Kong Special Administrative Region of China). However, the authors of this study pointed out that the Chinese population living alone in Hong Kong might be the one who have the capacity to do so [13]. Consequently, these individuals should not be classified as the most vulnerable ones.

These studies cited above focused on the association between social relations (explanatory variable) and falls (outcome variable) which appears plausible because, for example, loneliness might lead to functional limitations [14, 15], increasing the likelihood of falling. However, it also appears plausible that social relations can act as an outcome variable in this relationship. In sum, the association between social relations and falls might be bidirectional. Nevertheless, disentangling the complex coevolution of these variables is beyond the scope of the current cross-sectional study. In the current study, *experiencing a fall* in the preceding 12 months is considered as *explanatory variable* and *social relations* (i.e., social exclusion, loneliness and the number of important people in regular contact) are treated as *outcome measure*.

We hypothesize that falls are related to, in particular, subjective evaluations of social relations in terms of social exclusion and perceived loneliness. For example, falls might be associated with feelings of social exclusion or loneliness because individuals experiencing a fall might tend to avoid or be unable to engage in instrumental activities of daily living such as shopping or meeting friends and relatives. Because these individuals do not participate in popular activities, they might feel socially excluded or lonely. This appears plausible because previous studies [14–18] have demonstrated that functional limitations are associated with higher levels of loneliness and social exclusion. Please see the discussion section for further details.

Similarly, we hypothesize that falls are also significantly negatively associated to more objective measures of social relations such as the number of important people in regular contact. While it might be the case that the location of visits shift from, for example, meeting places in the city to home visits, we assume that the number of important people in regular contact decreases because fallers might tend to avoid various physical-related activities (e.g., cycling, walking, or go on a trip) and, consequently, at least some of the important people in regular contact might withdraw at higher levels of dependency. For example, it has been shown that falls can lead to a decline in balance, gait, and activities of daily living (ADL) functioning in community-dwelling older adults [19]. Moreover, another study among patients with neuromuscular disorders reported that falls are often accompanied by reduced activities [20]. In addition, falls can result in fear of falling [21]. Fear of falling in turn is associated with activity avoidance [22, 23].

Consequently, the aim of the current study was to examine whether falls are associated with social relations comprehensively. Thereby, a population-based sample of individuals aged 40 years and over in Germany was used. Knowledge concerning these associations would help to emphasize the meaning of these relationships. Moreover, this might underline the importance of fall interventions. Preventing falls in turn might help to prevent loneliness and social exclusion. This is important because loneliness and social exclusion are highly prevalent in old age [24]. Moreover, absence of loneliness and social exclusion are important for a good quality of life [25].

It is worth noting that the terms *social exclusion* and *loneliness* are often used synonymously in the literature [25]. However, they are distinct concepts [26]. For example, without feeling socially excluded, individuals can perceive themselves as lonely (and vice versa) [27]. Perceived social exclusion is the feeling that one does not belong to the society, whereas loneliness is the state that an individual’s social network is smaller than desired [24]. In addition, social exclusion was defined as a “multidimensional process of progressive social rupture, detaching groups and individuals from social relations and institutions and preventing them from full participation in the normal, normatively prescribed activities of the society in which they live” [28]. Indeed, loneliness and social exclusion are strongly correlated (e.g., in our study, pairwise correlation was *r* = .51, *p* < .001) but not equivalent. Further details are reported elsewhere [27, 29].

## Methods

### Sample

In this study, data were gathered from the German Ageing Survey (DEAS). This longitudinal cohort-based survey of community-dwelling adults aged ≥40 years in Germany started in 1996 (first wave). Follow-up waves comprising cross-sectional and panel samples took place in 2002 (second wave), 2008 (third wave), 2011 (fourth wave) as well as in 2014 (fifth wave). As recently stated by Klaus et al. [30] the main goal of the DEAS study is “to provide a representative national database containing information describing the living conditions of the country’s middle-aged and older population and to study diversity within the older section of the population, the process of ageing as it affects individuals and processes of social change as they relate to old age and ageing”. To this end, a cohort-sequential design was used, consisting of cross-sectional samples (baseline samples) and longitudinal samples. The cross-sectional samples included first time participants, whereas the panel samples included individuals already interviewed before. A two-stage sampling methodology was used in this study. Twelve thousand municipalities exist in Germany. Thereof, a random sample of 290 municipalities was drawn in 1996. The baseline samples have been stratified by gender, age and region. Individuals participating in the baseline assessment who provided written consent were contacted for future assessments. Klaus et al. [30] provided further details regarding the DEAS study.

In 2014, the response rate was 25% for the cross-sectional sample and it was 61% for the panel sample. The response rates of the German Ageing Survey are similar to other large German surveys [31]. Further details regarding sample selection bias are provided in the strengths and limitations section. Falls were not captured from wave 1 to wave 4. Thus, data for the current study were used from the fifth wave of the DEAS study (*n* = 7808 individuals provided data on experiencing a fall). All subjects provided written informed consent.

### Dependent variables

A short version [32] of the established 11-item De Jong Gierveld Loneliness Scale [33] was used to quantify loneliness (1 = “strongly agree”, 2 = “agree”, 3 = “disagree” to 4 = “strongly disagree”). Items are: “I miss having people around among which I feel comfortable”, “There are plenty of people I can rely on when I have problems”, “I often feel rejected”, “There are many people I can trust completely”, “I miss emotional security and warmth”, “There are enough people I feel close to”. It has been demonstrated that the scale used in our study is valid and reliable [34]. Scale represents the mean of at least three required valid items. In other words: If an individual only answered 2 or less loneliness items, no loneliness score was computed for this individual. Higher values reflect higher perceived loneliness. Cronbach’s alpha was .83 in our study.

Furthermore, the number of important people in regular contact (from 0 to 9) was assessed. The exact wording was: “We now want to look at people who are important to you and who you maintain regular contact with. These can include co-workers, neighbours, friends, acquaintances, relatives, and members of your household. Which people are important to you? If there are several, please just name the eight most important. Please give me these people’s first names and the first letters of their last names.” If the target persons wanted to name more than 8 persons, the network size was set to 9.

Moreover, social exclusion was measured using a scale, which was developed by Bude and Lantermann [35], consisting of four items (with a range from 1 = “strongly agree” to 4 = “strongly disagree”). Items are as follows: “I am worried to be left behind”, “I feel like I do not really belong to society”, “I feel that I am left out”, and “I feel excluded from society”. Scale represents the mean of at least 2 required valid items. Higher values reflect higher perceived social exclusion. Cronbach’s Alpha was .88 in the present study.

### Independent variables

#### Variable of interest: Falls

Using a common way of measuring the history of falls [36, 37], the experience of a fall in the preceding 12 months (yes; no) was assessed.

### Control variables

Various factors supposed to be important for social relations were included in the present study including socioeconomic factors, lifestyle factors, and self-rated health as well as morbidity [25, 26, 38–42]. Thus, control variables were included in the analyses as follows: age in years, sex, family status (married, and living together with spouse; others (married, and living separated from spouse; single; divorced; widowed), and individual monthly net equivalent income (OECD scale). Moreover, lifestyle factors were included: frequency of alcohol consumption and frequency of sports activities (both: ‘never’, ‘rarer than once a month’, ‘one to three times a month’, ‘once a week’, ‘several times a week’, and ‘daily’) as well as smoking behavior (daily smoker; casual smoker; former smoker; non-smoker). Self-reported height (meter) and weight (kg) was used to calculate body mass index (BMI) as weight divided by height-squared. Besides, the number of physical illnesses (cardiac and circulatory disorders; bad circulation; joint, bone, spinal or back problems; respiratory problems, asthma, shortness of breath; stomach and intestinal problems; cancer; diabetes; gall bladder, liver or kidney problems; bladder problems; insomnia; eye problems, vision impairment; ear problems, hearing problems; other illnesses or health problems) was included as control variable. For example, it has been shown that sociodemographic variables such as age are associated with the risk of falls [43]. Furthermore, it has been shown that lifestyle factors are associated with falls [44]. Moreover, various studies have demonstrated that chronic diseases were associated with an increased risk of falls [45].

As depressive symptoms might be another confounding variable, it was added to the main model in additional analysis. Depressive symptoms were quantified using Center for Epidemiological Studies Depression Scale (CES-D) [46] (short form, 15 items), ranging from 0 to 45 (high values indicate higher depressive symptoms).

In another regression model, the subscale ‘physical functioning’ of the SF-36 [47] was added to the main model, ranging from 0 (worst score) to 100 (best score).

### Statistical analysis

First, sample characteristics were displayed and pairwise correlations were computed. In addition, multiple linear regressions were used to model the relation of the outcome variable (loneliness, social exclusion, or the number of important people in regular contact) to falls in the preceding 12 months, controlling for several potential confounders. The model assumptions for linear regressions were checked. For example, in the linear regressions performed, it was tested for multicollinearity (using the variance inflation criterion). Across the regressions, it was found that the largest variance was 3.3, indicating that no problem with multicollinearity was present. In addition, the White test for heteroscedasticity in the error distribution was performed. Following the test statistics (with social exclusion as outcome measure: White’s general test statistic = 594.5, *p* < 0.001; with loneliness as outcome measure: White’s general test statistic = 369.3, *p* < 0.001; with number of important people in regular contact: White’s general test statistic = 248.5, *p* < 0.05), the null hypothesis of homoscedasticity was rejected. Consequently, robust standard errors were used. The normality assumption of the residuals were checked using normal-probability plots, showing that the residuals were approximately normally distributed.

In additional analysis, regressions were performed stratified by age (individuals younger than 65 years; individuals aged 65 years and above) to test whether the association between falls and outcome variables is age-specific.

The statistical significance was set at *p* < .05. Statistical analysis was performed using Stata 14.0 (Stata Corp, College Station, TX, USA).

## Results

### Description of the sample and bivariate correlations

In sum, 7808 participants (mean age: 64.5 years ± 11.2 years) reported whether they experienced a fall in the preceding 12 months or not. About half of these respondents were female (50.9%), more than two-thirds (70.1%) were married, living together with spouse. In total, 1372 respondents (17.6%) reported one or more falls in the past 12 months. The mean loneliness score was 1.78 (±0.54; ranging from 1 to 4), the mean social exclusion score was 1.60 (±0.59; ranging from 1 to 4), and the mean number of important people in regular contact was 5.23 (±2.70; ranging from 0 to 9). The average depressive symptoms score was 6.65 (±5.97; ranging from 0 to 44). The average ‘physical functioning’ score was 81.7 (±22.9, 0 to 100). Furthermore, descriptive statistics stratified by age group (< 65 years; ≥ 65 years) are depicted in Table 1.

**Table 1:** Characteristics, stratified by age group (< 65 years; ≥ 65 years) (*n* = 7808)

	Individuals <65 years (*n* = 3950)		Individuals ≥65 years (*n* = 3858)		*p*-value
	N/Mean	%/(SD)	N/Mean	%/(SD)	t-test/ chi^2^
Gender: Female	2139	53.8%	1836	46.2%	<.001
Age in years	55.0	6.3	73.8	5.9	<.001
Marital status: married and living together with spouse	2726	49.9%	2738	50.1%	.19
Monthly net equivalent income in Euro	2092.1	1490.8	1800.1	1240.6	<.001
Body-Mass-Index (BMI)	26.8	4.9	27.0	4.2	.08
Smoking status: Daily	805	75.1%	267	24.9%	<.001
- Yes, sometimes	217	70.7%	90	29.3%	
- Not anymore	1277	44.4%	1597	55.6%	
- Never been smoker	1541	44.2%	1948	55.8%	
Consumption of alcohol: Daily	378	40.4%	557	59.6%	<.001
- several times a week	1009	53.2%	888	46.8%	
- once a week	710	56.8%	540	43.2%	
- one to three times a month	524	55.3%	423	44.7%	
- less frequently	863	46.0%	1012	54.0%	
- never	369	41.5%	520	58.5%	
Physical activity: Daily	244	37.4%	409	62.6%	<.001
- several times a week	1161	54.5%	971	45.5%	
- once a week	740	51.9%	685	48.1%	
- one to three times a month	337	57.8%	246	42.2%	
- less frequently	575	62.7%	342	37.3%	
- never	801	38.2%	1296	61.8%	
Subjective health (from 1 = “very good” to 5 = “very bad”)	2.4	0.9	2.6	0.8	<.001
Number of physical illnesses	2.1	1.7	3.1	1.9	<.001
Experiencing a fall in the preceding 12 months	527	38.4%	845	61.6%	<.001
Social exclusion	2.6	0.6	2.6	0.6	.06
Loneliness	1.8	0.6	1.7	0.5	<.001
Number of important people in regular contact	5.5	2.7	5.0	2.7	<.001

Notes: Social exclusion was assessed using a scale developed by Bude and Lantermann (2006), ranging from 1 to 4 (higher values reflect higher perceived social exclusion); Loneliness was assessed using a short version (Gierveld and Van Tilburg 2006) of the De Jong Gierveld Loneliness Scale (Gierveld and Kamphuls 1985), ranging from 1 to 4 (higher values reflect higher perceived loneliness). Number of important people in regular contact: ranging from 0 to 9; Gender: Ref.: Male; Marital status: Ref.: married and living together with spouse; Smoking status: ranging from 1 = ‘daily’ to 4 = ‘never been a smoker’; Consumption of alcohol: ranging from 1 = ‘daily’ to 6 = ‘never’; Physical activity: ranging from 1 = ‘daily’ to 6 = ‘never’; Subjective health: ranging from 1 = ‘very good’ to 5 = ‘very bad’; Falls: Ref.: Not experiencing a fall in the preceding 12 months

Bivariate analysis (Table 2) revealed that falls were associated with social exclusion (*r* = .11, *p* < .001) and loneliness (*r* = .09, *p* < .001), whereas they were not related with the number of important people in regular contact. It is worth noting that while social exclusion is strongly associated with loneliness (*r* = .51, *p* < .001), both variables are weakly associated with the number of important people in regular contact (with social exclusion: *r* = −.08, *p* < .001; with loneliness: *r* = −.13, *p* < .001). In addition, all three outcome measures were significantly associated with marital status, income, alcohol consumption, physical activity, self-rated health, and the number of physical illnesses.

**Table 2:** Pairwise cross-sectional correlations (with Bonferroni-adjusted significance level; German Ageing Survey; fifth wave; *n* = 7808)

	Social exclusion	Loneliness	Number of important people in regular contact	Gender: Female	Age in years	Marital status: other marital statuses	Monthly net equivalent income in Euro	Body-Mass-Index (BMI)	Smoking status	Consumption of alcohol	Physical activity	Subjective health	Number of physical illnesses	Fall in the preceding 12 months
Social exclusion	1.00													
Loneliness	0.51^***^	1.00												
Number of important people in regular contact	−0.08^***^	−0.13^***^	1.00											
Gender: Female	0.03	−0.07^***^	0.07^***^	1.00										
Age in years	0.03	−0.05^**^	−0.12^***^	−0.10^***^	1.00									
Marital status: other marital statuses	0.10^***^	0.10^***^	−0.12^***^	0.15^***^	0.03	1.00								
Monthly net equivalent income in Euro	−0.17^***^	−0.10^***^	0.12^***^	−0.05^***^	−0.09^***^	−0.09^***^	1.00							
Body-Mass-Index (BMI)	0.07^***^	0.05^**^	−0.03	−0.11^***^	0.03	−0.01	−0.11^***^	1.00						
Smoking status	−0.05^**^	−0.08^***^	0.03	0.10^***^	0.22^***^	−0.09^***^	0.03	−0.01	1.00					
Consumption of alcohol	0.13^***^	0.05^**^	−0.09^***^	0.28^***^	0.03	0.12^***^	−0.17^***^	0.10^***^	0.03	1.00				
Physical activity	0.12^***^	0.07^***^	−0.15^***^	−0.07^***^	0.09^***^	0.05^**^	−0.17^***^	0.19^***^	−0.14^***^	0.11^***^	1.00			
Subjective health	0.24^***^	0.19^***^	−0.10^***^	−0.02	0.14^***^	0.04^+^	−0.16^***^	0.22^***^	−0.06^***^	0.15^***^	0.22^***^	1.00		
Number of physical illnesses	0.22^***^	0.17^***^	−0.05^**^	−0.04^*^	0.35^***^	0.06^***^	−0.14^***^	0.21^***^	0.03	0.09^***^	0.14^***^	0.44^***^	1.00	
Fall in the preceding 12 months	0.11^***^	0.09^***^	−0.01	0.08^***^	0.13^***^	0.06^***^	−0.03	0.06^***^	0.03	0.06^***^	0.04^+^	0.18^***^	0.23^***^	1.00

*** *p* < 0.001, ** *p* < 0.01, * *p* < 0.05, + *p* < 0.10. Social exclusion was assessed using a scale developed by Bude and Lantermann (2006), ranging from 1 to 4 (higher values reflect higher perceived social exclusion); Loneliness was assessed using a short version (Gierveld and Van Tilburg 2006) of the De Jong Gierveld Loneliness Scale (Gierveld and Kamphuls 1985), ranging from 1 to 4 (higher values reflect higher perceived loneliness). Number of important people in regular contact: ranging from 0 to 9; Gender: Ref.: Male; Marital status: Ref.: married and living together with spouse; Smoking status: ranging from 1 = ‘daily’ to 4 = ‘never been a smoker’; Consumption of alcohol: ranging from 1 = ‘daily’ to 6 = ‘never’; Physical activity: ranging from 1 = ‘daily’ to 6 = ‘never’; Subjective health: ranging from 1 = ‘very good’ to 5 = ‘very bad’; Falls: Ref.: Not experiencing a fall in the preceding 12 months; Categorical variables were treated as continuous variables for reasons of simplicity

### Regression analysis

Results of regression analysis are depicted in Table 3 (first column: with social exclusion as outcome measure; second column: with loneliness as outcome measure; third column: with number of important people in regular contact as outcome measure).

**Table 3:** Determinants of loneliness, social exclusion and number of important people in regular contact. Results of multiple linear regression analysis (German Ageing Survey, fifth wave)

	(1)	(2)	(3)
	Social exclusion	Loneliness	Number of important people in regular contact
Gender: Female (Ref.: male)	−0.0106	−0.122***	0.466***
	(0.0147)	(0.0136)	(0.0674)
	[−0.00901]	[−0.113]	[0.0866]
Age in years	−0.00380***	−0.00665***	−0.0241***
	(0.000701)	(0.000653)	(0.00313)
	[−0.0721]	[−0.137]	[−0.100]
Marital status: other marital statuses (Ref.: married and living together with spouse)	0.0728***	0.114***	−0.635***
	(0.0159)	(0.0145)	(0.0688)
	[0.0569]	[0.0964]	[−0.108]
Monthly net equivalent income in Euro	−4.59e-05***	−2.33e-05***	0.000148***
	(5.51e-06)	(4.80e-06)	(2.34e-05)
	[−0.109]	[−0.0602]	[0.0767]
Body-Mass-Index (BMI)	−0.00311+	−0.00428**	0.00537
	(0.00165)	(0.00145)	(0.00703)
	[−0.0242]	[−0.0362]	[0.00916]
Smoking status: yes, sometimes (Ref.: daily)	−0.0768*	−0.0758*	0.239
	(0.0355)	(0.0377)	(0.184)
	[−0.0256]	[−0.0274]	[0.0174]
not anymore	−0.0177	−0.00926	0.152
	(0.0235)	(0.0213)	(0.102)
	[−0.0145]	[−0.00826]	[0.0274]
never been a smoker	−0.0342	−0.0495*	0.109
	(0.0230)	(0.0211)	(0.101)
	[−0.0290]	[−0.0454]	[0.0201]
Consumption of alcohol: several times a week (Ref.: daily)	0.00646	−0.0194	0.130
	(0.0220)	(0.0213)	(0.108)
	[0.00475]	[−0.0154]	[0.0208]
once a week	0.0200	−0.00813	0.0311
	(0.0248)	(0.0236)	(0.118)
	[0.0124]	[−0.00546]	[0.00420]
one to three times a month	0.0604*	−0.00932	0.0233
	(0.0264)	(0.0253)	(0.127)
	[0.0337]	[−0.00564]	[0.00284]
less frequently	0.0836***	0.0160	−0.245*
	(0.0246)	(0.0230)	(0.111)
	[0.0606]	[0.0125]	[−0.0388]
never	0.132***	0.0548*	−0.548***
	(0.0310)	(0.0275)	(0.131)
	[0.0710]	[0.0319]	[−0.0646]
Physical activity: several times a week (Ref.: daily)	−0.0287	−0.0104	−0.0582
	(0.0252)	(0.0249)	(0.126)
	[−0.0218]	[−0.00857]	[−0.00963]
once a week	0.00542	0.00899	−0.334*
	(0.0269)	(0.0261)	(0.132)
	[0.00357]	[0.00641]	[−0.0481]
one to three times a month	0.0323	0.000671	−0.0156
	(0.0327)	(0.0311)	(0.159)
	[0.0146]	[0.000327]	[−0.00153]
less frequently	−0.00515	0.0127	−0.415**
	(0.0294)	(0.0288)	(0.144)
	[−0.00283]	[0.00755]	[−0.0497]
never	0.0319	−0.00927	−0.763***
	(0.0271)	(0.0259)	(0.128)
	[0.0240]	[−0.00757]	[−0.126]
Subjective health (from 1 = “very good” to 5 = “very bad”)	0.0990***	0.0826***	−0.149***
	(0.0102)	(0.00915)	(0.0434)
	[0.140]	[0.127]	[−0.0462]
Number of physical illnesses	0.0449***	0.0390***	0.0731***
	(0.00468)	(0.00403)	(0.0197)
	[0.143]	[0.134]	[0.0509]
Fall in the preceding 12 months (Ref.: no)	0.0816***	0.0772***	0.0870
	(0.0195)	(0.0176)	(0.0848)
	[0.0525]	[0.0537]	[0.0122]
Constant	2.595***	2.099***	6.813***
	(0.0730)	(0.0667)	(0.325)
Observations	7026	6997	7093
R^2^	0.108	0.090	0.070

Comments: Beta-Coefficients are reported; Cluster-robust standard errors in parentheses. Standardized beta-coefficients in square brackets; *** *p* < 0.001, ** *p* < 0.01, * *p* < 0.05, + *p* < 0.10. Observations with missing values were dropped (listwise deletion). Loneliness was assessed using a short version (Gierveld and Van Tilburg 2006) of the De Jong Gierveld Loneliness Scale (Gierveld and Kamphuls 1985). Social exclusion was assessed using a scale developed by Bude and Lantermann (2006)

Controlling for potential confounders, linear regression analysis showed that reporting a fall in the previous 12 months was associated with higher social exclusion scores (β = .08, *p* < .001), and higher loneliness scores (β = .08, *p* < .001). Contrarily, reporting a fall in the preceding 12 months was not associated with the number of important people in regular contact.

Moreover, all outcome measures were associated with age, marital status, income, self-rated health, the number of physical illnesses, and alcohol consumption (daily vs. never).

In additional analysis, regressions were performed stratified by age (results not shown, but available upon request). In sum, experiencing a fall was significantly associated with social exclusion (individuals younger than 65 years: β = .13, *p* < .001; individuals aged 65 years and above: β = .06, *p* < .05) and loneliness (individuals younger than 65 years: β = .10, *p* < .001; individuals aged 65 years and above: β = .06, *p* < .01) in both age brackets, whereas it was not associated with the number of important people in regular contact in these age brackets (individuals younger than 65 years: β = .06, *p* = .66; individuals aged 65 years and above: β = .11, *p* = .34).

In further additional analysis, depressive symptoms were added to the main model. Linear regression analysis revealed that the association between reporting a fall and social exclusion (β = .05, *p* < .01) as well as loneliness (β = .05, *p* < .01) remained almost the same. In addition, the association between reporting a fall and the number of important people in regular contact remained non-significant.

In another model, physical functioning was added to the main model. Regression analysis showed that the association between reporting a fall and social exclusion (β = .07, *p* < .001) as well as loneliness (β = .08, *p* < .001) remained virtually the same. Moreover, the link between reporting a fall and the number of important people in regular contact remained non-significant.

## Discussion

### Main findings

Using data from a representative sample of individuals in the second half of life in Germany, the current study aimed to examine the relationship between falls and social ties, covering loneliness, social exclusion and the number of important people in regular contact. In total, 17.6% of the individuals reported to have one or more falls in the past 12 months. Controlling for various possible confounding variables, linear regressions showed that falling in the past 12 months was associated with higher social exclusion, and increased loneliness, whereas it was not associated with the number of important people in regular contact. Besides, all outcome measures were significantly associated with age, marital status, income, self-rated health, and the number of physical illnesses.

### Relation to previous studies

To our best knowledge, only a few studies investigated the association between falls and social relations before. Please see the introduction for further details. Most of the existing studies showed an association between social factors such as living alone and falls.

The results of the current study emphasize the association between falls and feelings of loneliness as well as social exclusion, whereas falls were not associated with the more objective measure of number of important people in regular contact. A possible explanation might be that while experiencing a fall is strongly associated with a change in the perception of social ties, falls are not associated with actual changes in social relations among older adults. The first assumption (change in perception of social ties) might be explained by the fact that falls are associated with various factors such as physical activity, self-esteem, health-related quality of life, anxiety or depressive symptoms [5–9]. These factors are in turn associated with feelings of loneliness and social exclusion [48–50]. In addition, friends and acquaintances might come to support the individual experiencing a fall. Thus, the individual in need for care might feel guilty and unable to repay the support received. Consequently, an adult who fell might experience most social relations as unidirectional, resulting in dissatisfaction with the quality of the relationship. This ultimately might lead to feelings of social exclusion and loneliness.

The non-significant relationship between the number of important people in regular contact and falls might be explained by the fact that important people in regular contact might maintain their visits (e.g., home visits; regular phone calls). Thus, it might be the case that the location of visits changes. Moreover, the quality of visits (e.g. duration of visits, personal contact; important people might stop performing certain activities during visits as the individual experiencing a fall is no longer willing to engage in such activities) might decrease, which should be investigated in future studies. However, these factors do not affect the variable number of important people in regular contact.

It is worth acknowledging that the partial η^2^ for these two explanatory variables was .003 each (0.3% of the variability in social exclusion and loneliness explained) in the models displayed in column 1 and column 2 of Table 3. Consequently, the clinical relevance might be quite small.

Summarizing, using a representative sample of community-dwelling older individuals in Germany, the present study provided first insights into the association between falls and social ties. It demonstrates that it is worth studying the relationship between falls and social ties in a broader sense.

### Strengths and limitations

This is the first study that investigates the association between falls and social relations including loneliness, social exclusion and the number of important people in regular contact. The German Ageing Survey is a large, population-based study of community-dwelling subjects aged 40 and over. Loneliness and social exclusion were operationalized using established scales. In the regression model, various potential confounders were considered.

Generalization of our findings to, for example, individuals with low education might be limited for reasons of sample selection bias, though it has been demonstrated that selectivity effects are small in the German Ageing Survey [30] and the distribution of sociodemographic factors including family status, household size, family composition is close to the distribution within the German population [51]. Individuals reported whether they had at least one fall in the preceding 12 months (dichotomous; without reporting the quantity of falls), thus a recall bias may have occurred. Nevertheless, we strongly believe that individuals generally recall a fall in the past year as this might be an important event. It is worth noting that no additional information on falls were provided in this dataset (e.g., severity of the falls or frequency of falls).

Moreover, which is worth emphasizing, this is a *cross-sectional* study. The causal direction of this relationship (falls and social relationships) could be argued to be reciprocal. Longitudinal studies are required to gain insights into the causal relationship between falls and social relations, adjusting for time-constant factors.

## Conclusion

Findings stress the relation between falls and feelings of loneliness and social exclusion, whereas falls were unrelated to the more objective measure of number of important people in regular contact, suggesting that falls are related to particularly subjective measures of social relations. Consequently, interventions aiming at preventing falls in older age might contribute to diminish feelings of loneliness or social exclusion.

## Abbreviations

ADL: Activities of daily living
BMI: Body mass index
DEAS: German Ageing Survey
OECD: Organization for economic co-operation and development
